# Perspective: Food Access at Dollar Stores and Its Implications for Public Health—Report of a Workshop on Identifying Research Priorities

**DOI:** 10.1016/j.advnut.2024.100319

**Published:** 2024-10-11

**Authors:** Wenhui Feng, Hailey Fromkin, J Becket Harney, Ryan Evans, Colin M Gerrity, Sean B Cash

**Affiliations:** 1 Tufts University School of Medicine, Department of Public Health and Community Medicine, Boston, MA, USA; 2 Tufts University Gerald J. and Dorothy R. Friedman School of Nutrition Science and Policy, Boston, MA, USA; 3 Tufts University, Technology Services, Medford, MA, USA

**Keywords:** dollar stores, food access, food retail, public health, diet quality, consumer behavior

## Abstract

The rapid growth of dollar stores as retail sources of food in the United States is a phenomenon with implications for diets, nutrition, and well-being. We convened a broadly interdisciplinary group of researchers and experts from government and academia at the 2-day Food Access at Dollar Stores (FADS) workshop, held in Boston, MA in 2022. The event brought together economists, social scientists, public health researchers, and advocates to discuss the concerns and research questions raised by the growth of dollar stores and their increased role in food retail and access. In-person, moderated discussions on day 2 of the workshop generated a range of topics considered important for future research. A subsequent survey, using a modified Delphi approach, identified priority research areas. Nine research area categories emerged as a result of discussion at the FADS workshop and received prioritization from the experts: Local community impacts; Health and nutrition impacts; Policy and programs; Systemic issues – racism, poverty, and food access; Store offerings and locations; Shoppers and customers; Employees and employment; Corporate distribution, strategy, and marketing; and Dollar stores compared with other food sources. The growth of dollar stores as food retailers remains an under-researched area of study for food access and nutrition that requires interdisciplinary expertise and collaboration to understand.

**Statement of Significance**

Dollar stores have grown rapidly as food retailers in the United States over the past decade. This work reports the findings of a research workshop and provides a roadmap for prioritizing research topics and tools related to understanding the public health, equity, and economic impacts of this important change in many consumers’ access to food.

## Introduction

Dollar stores are a rapidly expanding source of food for Americans, but there is a need for further research on these retailers’ impacts across a variety of metrics and disciplines. From 2008 to 2020, dollar stores were the fastest-growing food retailers in terms of household-level spending, particularly in rural areas [1]. Despite their growing importance for the American food consumer, relatively little scholarship exists on dollar stores. Research to date has largely focused on the geographic distribution of dollar stores [2] and their relationship with other food retailers [3,4]. Experts across multiple disciplines (for example, geography [5], policy and law [6], economics [2,3,7], and public health [1]) recognize the need to better understand the implications of dollar stores for food access, the dietary health of rural and other underserved communities, and dollar stores’ role in the economic well-being of the communities in which they are located.

Dollar stores have attracted attention from both advocacy groups and policy-makers. The Institute for Local Self-Reliance has published multiple reports and toolkits focusing on dollar stores’ impact on communities and local grocery stores [8,9]. The Center for Science in the Public Interest (CSPI) recently issued a report highlighting the nationwide increase in dollar stores and urging interventions to encourage healthier offerings [10]. Recent research findings have informed – but not yet settled – the debate over whether and how dollar stores may be impacting the larger competitive landscape for food retail on a national basis [2,3,7]. In response to fears that dollar stores limit access to healthy foods or potentially harm local business, ≥25 municipalities across the country (including locations in Alabama, Florida, Georgia, Ohio, Oklahoma, South Carolina, and Texas) have imposed restrictions on the expansion of new dollar stores, including limitations on the geographic density of dollar stores and incentives for dollar stores to designate larger in-store areas for fresh produce [6]. At the national level, the United States Department of Agriculture updated its Supplemental Nutrition Assistance Program (SNAP) retailer eligibility standards to require SNAP-authorized stores to provide a wider variety of food items as of 2018 [7 CFR 231.2 and 7 CFR 278.1(b)(1)(ii)(A)]. This stocking requirement targeted dollar stores, gas stations, and other retailers that have attracted concern for having less diverse food offerings.

Despite this policy interest in dollar stores, there is not yet sufficient evidence to inform policy-makers on the role of dollar stores. Although anecdotes have driven policy and discussion around dollar stores [11], much of the evidence base for dollar stores is still in development. For example, recent considerations of the impacts of dollar stores on other store formats have returned mixed results. Chenarides et al. [12] found supermarkets and other larger-format stores thrive as dollar stores expand, although other small-format stores (such as convenience stores) suffer [2]. On the contrary, Caoui et al. [3] and Lopez et al. [7] found dollar store expansion led to a decline in grocery stores. A case study in Tompkins County, NY that followed both quantitative and qualitative methods found that closures of local grocers due to competition from Dollar Stores had negative effects on former employees and vulnerable community members [13]. It is also not clear if, despite the limited offerings at many dollar stores, families with increased levels of food expenditures in dollar stores use what they save to purchase healthier food items elsewhere – leaving the overall healthfulness of household food purchases unknown. To prioritize research needs in this field, subject matter experts were convened following a modified Delphi method both for moderated in-person discussions and a follow-up survey to elicit recommendations and research priorities.

### Food access at dollar stores workshop

The Workshop on Food Access at Dollar Stores (FADS) and its Implications for Public Health was hosted by the Gerald J. and Dorothy R. Friedman School of Nutrition Science and Policy at Tufts University in 2022. The event was supported by the Agriculture and Food Research Initiative conference grant program, grant no. 2022-67023-36955 from the United States Department of Agriculture. The workshop was co-hosted by Dr. Sean B. Cash of the Friedman School of Nutrition Science and Policy, Tufts University, and by Dr. Wenhui Feng, from the Tufts University School of Medicine. The scientific committee responsible for reviewing and selecting the featured speakers included Dr. Lauren Chenarides (now in the Department of Agricultural and Resource Economics at Colorado State University); Dr. Chelsea Singleton of the School of Public Health & Tropical Medicine, Tulane University; and Dr. Ricky Volpe of the College of Agriculture, Food & Environmental Sciences at California Polytechnic State University. The overall goal of this workshop was to facilitate discussions on the state of the science relating to dollar stores to identify priorities for future research.

This 2-day workshop took place in person and brought together 61 attendees, primarily researchers interested in dollar stores and their roles in food access, including economists, public health researchers, geographers, and other social scientists. A major goal was to include work across broad disciplinary approaches. Attendees discussed the concerns and questions raised by the growth of dollar stores, and opportunities presented by dollar stores’ increased role in food retail and access. Through research panels, poster presentations, and networking sessions on day 1, and facilitated discussions on day 2, this event fostered interdisciplinary collaborations to collectively review and frame the research priorities regarding the role of dollar stores in food access, dietary quality, and community livelihoods. A full list of presenter information by panel as well as information on presenters featured in the poster showcase on day 1 and proceedings of the event can be found in the supplementary material.

## Methods

Across the 2-day workshop, attendees were engaged in collectively developing an “investigatory landscape” of research priorities including unaddressed research questions, existing and needed data sources, and opportunities for interdisciplinary collaboration. Colin Gerrity and Ryan Evans of the Tufts Design Services team led the overall collaboration, both bringing experience in facilitating workshops with multiple stakeholders to inform decision-making. Engagement took place in 2 phases, using a model of divergent and convergent ideation adapted from Design Thinking frameworks. [14] Day 1 consisted of 5 panels: regulation; nutrition and health; geography and industrial organization; the changing role of dollar stores; and community perspectives. Following each panel discussion, attendees were asked to respond to 3 prompt questions:

• What are the most important unaddressed research questions that come to mind in relation to this panel discussion?
• What new data sources will be needed to expand on the work described in this session?
• What research disciplines will need to collaborate to make progress in this session’s topic areas?

Responses to the questions were gathered using Poll Everywhere, a lightweight real-time survey tool [15]. Over 190 responses were received (100+ research questions, 60+ data source needs, and 30+ research disciplines) on the first day. Responses were organized into themes by the workshop facilitators and then presented to attendees for consideration on day 2 in moderated plenary discussion sessions. These discussions aimed to further develop and refine the identified research priorities and needs. Engagement on day 2 culminated in a plenary discussion that asked attendees to organize the responses into 5 topics, mirroring the topics of the panel discussions on day 1. Attendees were asked to further detail each topic by identifying a set of key enablers and barriers to collaboration.

The organizing committee captured research priority responses and topics via poll and discussion during the 2-day workshop and distributed these later to attendees for rank ordering. This was implemented through a post-workshop survey following a modified Delphi approach from November 2022 through January 2023. The Delphi method is an effective tool to anonymously elicit prioritization of research areas from a group of experts [16]. The method poses questions to experts individually over multiple rounds, seeking to develop a consensus. In a standard Delphi approach, experts do not communicate with one another and are typically surveyed over 3 or more rounds, each time provided with new information summarized from the responses of the full group. We apply a “modified Delphi approach”: experts worked collaboratively for the first round, and a total of 2 rounds were conducted. For the purposes of this study, the initial results from day 2 of the workshop were considered the first round of elicitation, establishing the research areas that would later be prioritized. The follow-up elicitation was sent as a survey to rank and assess key priorities for the research needed in this area after participants had time to independently reflect on their discussion. The results were then analyzed to identify leading research needs.

The population included in the study consisted of experts who attended the FADS Workshop (including those who had planned to attend but were unable to travel due to COVID-19 or other last-minute concerns). All workshop attendees (other than the 2 co-hosts) were sent an email (*n* = 59) asking them to complete the survey. We received 30 responses, from 14 academic faculty members, 2 research staff members, 3 staff members at non-academic institutions, 1 staff member from an advocacy organization, and 10 graduate students. Participants reported their primary discipline affiliation, totaling 12 economics specialists, 8 nutrition, 5 public health, 1 law, and 1 childhood food insecurity specialist.

Participants completed a simple ranking of insights first shared at the workshop. Analysis was limited to the generation of descriptive summaries using simple ranking approaches, where participants were asked to identify the 3 most important broad areas for future research, as well as the 2 most important specific topics (that is, research questions) within each of the 9 areas of inquiry. Responses were tabulated to determine the proportion of experts responding to each category who prioritized a given specific topic for future research. Finally, participants were asked to provide open-ended input on what new data sources will be needed and identify stakeholders whose collaboration will be necessary to expand dollar store research.

## Results

We present summary results from the FADS workshop elicitation exercises and a follow-up survey in Table 1 and Table 2. Table 1 reports the results of participants selecting their top 3 most important categories for research. *Health/Nutrition Impacts* received the greatest number of votes, followed by *Policy/Programs* and *Systematic Issues* tied for second. Table 2 indicates the prioritization of specific research questions and topics within categories. Across the 9 broad categories of unaddressed research areas, the attendees gave certain topics and potential research questions greater priority. Although some categories received clear prioritization for research, others returned more varied responses reflecting a variety of different directions one might take. Cross-cutting research needs were also identified, speaking to the need for interdisciplinary research approaches. The categories that received the clearest prioritization were *Local Community Impacts*, *Policy and Programs*, and *Employees/Employment*. The categories that resulted in more heterogeneous responses were *Shoppers/Customers* and *Corporate Strategy*.

**TABLE 1:** Prioritization of research categories.

Research category	Votes received
Local community impacts	14
Health/nutrition impacts	18
Store offerings/locations	6
Dollar store vs. other food sources	7
Employees/employment	2
Corporate distribution/strategy/marketing	3
Systematic issues – racism, poverty, and food access	15
Shoppers/customers	6
Policy/programs	15

Participants were asked to identify the 3 most important (unordered) categories of research regarding dollar stores *n* = 29.

**TABLE 2:** Prioritization of unaddressed research question topics by category, ranked by proportion of respondents indicating each question as a priority.

**Local community impacts**	
Community needs and wants	0.68
Local economics	0.57
Community coalition building with dollar stores/food retailers to offer healthier, but still affordable food options	0.57
Community relationships with dollar stores vs. smaller food retailers	0.25
**Health/nutrition impacts**	
Health and socio-environmental impact of fresh produce at dollar stores	0.57
Impact of healthier dollar store offerings and household behavior change	0.46
Connection between SNAP acceptance at dollar stores and poor nutrient density	0.36
More health outcome measures linkable to scanner data	0.36
Possibility of community nutrition education boosting demand for nutritious food purchases at dollar stores	0.25
Community coalition building with dollar stores/food retailers to offer healthier, but still affordable food options	0.21
**Store offerings/locations**	
Locations of neighborhoods where dollar stores offer fresh produce compared to "conventional" dollar stores	0.69
Difference in getting fresh produce from various venues like dollar stores, supermarkets, farmer's markets, etc.	0.48
Consistency or inconsistency of what food is available at individual dollar store locations	0.41
Regional differences in the big 3 dollar stores’ stocking and entry strategies	0.41
**Dollar store vs. other food sources**	
Differences between dollar store market basket prices and those of other food retail stores	0.58
Dollar store and convenience store comparison	0.50
Similarity of food products sold at dollar stores to products offered through federal commodity programs or food banks	0.42
Impact of dollar store entry on food bank use	0.27
Dollar stores vs. fast-food restaurants. Impact of dollar store entry on food bank use	0.23
**Employees/employment**	
Dollar store impact on wage	0.78
Care being provided to employees in dollar stores/impact on health equity	0.67
Similarities of portion of dollar store employees to Walmart staff on SNAP	0.56
**Corporate distribution/strategy/marketing**	
HEI of dollar store food offerings	0.44
Shifting subsidies: how agricultural financial support perpetuates cheap unhealthy food	0.40
Role of food and beverage brands: pay in slotting fees/making special projects for the dollar store market	0.24
Impact of dollar stores not selling any food; upstream corporate dynamics that influence nutrition marketing in dollar stores/ the influence on customer food choice	0.20
Readiness for organizational change among dollar store c-suite representatives	0.20
Format-store loyalty and store switching: what does customer loyalty mean to dollar store chains and how does it compare to store loyalty at retail grocers?	0.16
Likelihood of fresh produce sold at dollar stores going to homes vs. landfills (food waste)	0.12
Cost data from dollar store chains	0.12
Dollar stores inability to sell products given their distribution structures	0.08
Dollar stores creating a product liability by offering fresh produce at a similar quality scale as their other offerings	0.04
**Systemic issues - racism, poverty, and food access**	
Systematic and structural racism in food access for food-insecure urban and rural communities	0.59
Dollar stores potentially profiting off of federal systemic inadequacies	0.52
High demand for dollar stores vs. high supply of poverty	0.41
Role of dollar stores in the perpetuation of neighborhood disorder, blight, and crime	0.31
Systemic issues are enabling dollar store success	0.17
**Shoppers/customers**	
Willingness of dollar store customers to pay more for healthier options	0.44
Differences or similarities in how dollar store shoppers make their food purchasing decisions	0.44
Cumulative impacts suffered by dollar store shoppers when compared to other retail shoppers	0.41
Community and consumer perception of dollar stores, and how that varies by region	0.37
Individual-level commuting and other travel patterns linkable to food acquisition	0.33
**Policy/programs**	
Policy/legal considerations for mandating or incentivizing healthier options at dollar stores	0.79
SNAP minimum stocking requirements at dollar stores	0.48
Farm bill contribution to food access/SNAP/WIC at discount retailers	0.34
Welfare effects	0.24
WIC stockage	0.14

Abbreviations: HEI, Healthy Eating Index; SNAP, Supplemental Nutrition Assistance Program; WIC, Special Supplemental Nutrition Program for Women, Infants, and Children.

### Local community impacts

Within the local community impacts category, 1 topic area received clear prioritization: “community needs and wants.” Two topics (“local economics” and “community coalition building”) tied for second place prioritization within this category, although “community relationships with dollar stores compared to other small food retailers” received a distant fourth place prioritization. Some specific research questions proposed for this category included “What is the impact of Dollar Store entry in low-income markets?” and “Will the entry of dollar stores reduce the number of grocery stores and other stores?”

### Health and nutrition impacts

One research area received significant prioritization in the health and nutrition category: “Health and socio-environmental impacts of fresh produce at dollar stores.” A secondary priority in this category was understanding the impact of healthier dollar store offerings and potential for household behavior change.

### Store offerings/locations

One prominent research area emerged in this category. The prioritized research area focuses on mapping the locations where dollar stores have begun to stock fresh produce compared to traditional dollar stores that do not. Survey respondents suggested that research using GIS may effectively reveal some trends.

### Dollar stores compared with other food sources

This category sought to compare dollar stores with other vendors from which consumers may purchase food. The leading area prioritized for research was an assessment of the differences between dollar store market basket prices and those of other food retail stores. Market baskets are a commonly used tool in economics to track a selected a selection of goods across time or contexts. Ranked closely behind was a comparison with convenience stores, although comparisons to food bank offerings and fast-food locations received less focus. One suggested method to conduct this comparison that received great prioritization was the use of market basket prices.

### Employees/employment

One research area received significant prioritization: “the impact of dollar stores on wage.” One method raised in the survey to explore this was comparing the enrollment in SNAP and other food assistance programs among dollar store employees against other food retail workers.

### Systemic issues – racism, poverty, and food access

A clear research area prioritized in this category seeks to understand the systematic and structural racism in food access for food-insecure urban and rural communities. A secondary research area seeks to explore the ways that racial and economic inequality may contribute to dollar store profits.

### Corporate distribution, strategy, and marketing

This category returned several closely ranked research areas, with no specific area dominating prioritization. A highly prioritized research area was the need for additional metrics to assess store-level/retailer healthfulness. Although experts noted that the Healthy Eating Index is currently used to assess alignment with the Dietary Guidelines for Americans, this metric cannot be easily applied to evaluate if one retailer overall offers more healthful food than another. Additional research areas included “the role of agricultural subsidies in supporting the prevalence of low-cost, highly processed food” and “research into the impact of food and beverage brands paying slotting fees to receive prominent displays for packaged goods.”

### Shoppers/customers

The expert panelists were less consistent in their prioritization for this category. The leading research areas included explorations of dollar store shoppers’ willingness to pay premiums for healthier options, the cumulative impacts experienced by dollar store shoppers compared to those who shop primarily at other retail locations, and research into the food purchasing decision processes among dollar store food shoppers. A specific research question that ranked highly within this category was “How does dollar store entry affect consumer spending?”

### Policy and programs

One research topic dominated in prioritization within the policy and programs category: “Policy and legal considerations for mandating or incentivizing healthier options at dollar stores.” As local municipalities have enacted legislation/policies to this effect, there is a great opportunity to identify the factors that impact their success or obstacles. The research focused on SNAP minimum stocking requirements also received prioritization. Some specific research questions that were highly ranked in this category include: “how does dollar store pricing change once stores begin accepting SNAP?”; “How are zoning laws affecting dollar store entry?”; and “How do local dollar store policies change consumption behavior of the local community?”

## Discussion

Research investigating the priority areas established in this survey is already underway. As noted above, additional research exploring the tensions between these findings and assessing what community-level conditions may lead to different outcomes would benefit both the research community and policy decision-makers.

The priority research areas identified will continue to benefit from approaches across disciplinary boundaries. Relevant to the topic area of dollar store employees and employment, ethnographic research in the field of sociology has considered the experiences of reported experiences of surveillance [17]. Developing teams of researchers that can bring different perspectives to questions surrounding dollar stores was an explicit goal of the FADS workshop. To the best of our knowledge, at least two new teams of investigators that met at this workshop have gone on to submit research grants on dollar-store-related topics at the time of this writing.

In addition to the research areas prioritized through the FADS workshop, community advocates have continued to call for additional research into dollar stores. The CSPI released a report in October 2023 which included research recommendations. The 4 areas of research priorities identified were *1*) local-level surveys to capture consumer perceptions that might inform public policy, *2*) research into geographic variation in dollar store food offerings across the country, *3*) collaborations with dollar store corporate offices to review internal research findings, and *4*) pilot studies of healthy food marketing interventions in dollar stores [10]. This call for research from a leading consumer advocacy organization matches many of the prioritized research topics that surfaced in our survey.

Since the 2022 conference, some progress has been made in researching this field. Our research team has conducted a literature review that is underway. Notable work includes further research into the effects of dollar store entry on local grocers as in Chenarides [12], which found that independent and small-format grocers exit once dollar stores enter an area, but large-format grocers such as supermarkets and supercenters are unaffected. This evidence aligns with Lopez’s [7] findings that independent grocery retailers contract in response to dollar store entry. Furthering understanding of consumers’ perceptions of dollar stores, the CSPI October 2023 report surveyed 750 people who live near a dollar store [10]. They reported a positive perception of dollar stores among community members – 82% of respondents indicated that dollar stores helped their community. An additional 81% of respondents indicated that dollar stores should stock more healthy options.

The workshop and survey, although the first on this topic, did have notable limitations. First, this event was not a comprehensive set of stakeholders. Focusing explicitly on academics and community advocate participants meant that industry and regulatory stakeholders were not present. This decision was intentionally made to allow for open communication on research priorities and research in progress. The advocates who joined were aware that the aims of this event centered on research first. Additionally, despite the workshop having funding support that allowed the organizers to provide accommodations for all attendees, travel expenses could not be covered by the organizers, and as such some potential participants may not have been able to attend.

The stated goal of the FADS workshop was to serve as a catalyst for this fledgling research topic and spark interdisciplinary assessments of dollar stores’ role in the changing United States food retail landscape. By combining valuable in-person discussion to capture real-time responses and challenges to the proposed research and following with a modified Delphi survey, this article seeks to present a roadmap for future academic inquiry into this topic.

In conclusion, the conference participants voiced excitement at the prospect of this shared research focus and emphasized the need for a greater evidence base and literature surrounding dollar stores. The modified Delphi approach following the FADS workshop provides a clear roadmap for research priorities in the fields of economics, nutrition, and public health focused on dollar stores. The research focused on local community impacts, assessment of policy and programs, and dollar store employee wages received the strongest prioritization from the panel of experts. Establishing robust scientific baselines may inform public policy to support low-income Americans and future inquiry into this expanding retail category.

## Author contributions

The authors’ responsibilities were as follows – SBC, WF: responsible for conceptualization, management, and funding of the workshop; SBC, RE, CMG, WF: responsible for design of the expert elicitation and data collection; SBC, HF, JBH, WF: responsible for data analysis; JBH: led the first draft of the report with input from all authors; and all authors have reviewed and approved the manuscript.

## Conflict of interest

SBC and WF report financial support was provided by the National Institute of Food and Agriculture (USDA). All other authors declare that they have no known competing financial interests or personal relationships that could have appeared to influence the work reported in this paper.

## Funding

This work is supported by the Agriculture and Food Research Initiative (AFRI) conference grant program, Grant No. 2022-67023-36955 from the USDA National Institute of Food and Agriculture. Partial funding for open access was provided by Tufts University Hirsh Health Sciences Library's Open Access Fund.
