# Supplementary material for: Perspective: Food Access at Dollar Stores and Its Implications for Public Health—Report of a Workshop on Identifying Research Priorities
Source: Adv Nutr. 2024 Oct 11;15(12):100319. doi: 10.1016/j.advnut.2024.100319 (PMC11705570; doi:10.1016/j.advnut.2024.100319)
Supplement: Multimedia component 1 [file mmc1.docx]

Supplemental – Food Access at Dollar Stores Workshop Presenter Information

**Panels**:

Research Panel 1 – Regulation

Moderator: Parke Wilde, Tufts University

*The Impact of Dollar Store Expansion on Local Market Structure and Food Access* Presenter: Matthew Osborne, University of Toronto Co-authors: El Hadi Caoui and Brett Hollenbeck

*Local Measures to Limit Dollar Stores' Spread: A Policy Scan*

Presenter: Julia McCarthy, New York State Health Foundation

Co-author: Chelsea Singleton

*Food Access at Dollar Stores - Framework Analysis (CSR, ESG, and Beyond)*

Presenter: Darin Detwiler, Northeastern University

Research Panel 2 – Nutrition and Health

Moderator: Sean Cash, Tufts University

*Impact of Dollar Stores on Household Shopping Patterns and Nutrition*

Presenter: Erik James, Wharton School of the University of Pennsylvania

*Obesity for a Dollar: What a Bargain!*

Presenter: Sandro Steinbach, University of Connecticut

Co-authors: Qianxia Jiang and K. Cooksey Stowers

*Consequences of the almighty dollar [store]: A Regional Nutrition Environment Survey*

Presenter: Bree Bode, Wayne State University

Co-authors: Rachael D. Dombrowski, Kathryn Gray-Knoff, Noel Kulik, Hadis Daztgerizad, Kibibi Blount-Dorn, Winona Bynum, and Jane Kramer

| **Research Panel 3 - *Geography and Industrial Organization***  *Dollar Store Expansion, Food Retail Competition, and Rural Employment* Presenter: Keenan Marchesi, USDA Economic Research Service Co-authors: Sandro Steinbach and Enrique A. Figuero  *The Geography of Dollar Stores* Presenter: Chuck Grigsby, University of Florida Co-authors: Conner Mullally and Richard Volpe   *Dollar Store Entry and Rural Welfare* Presenter : Lauren Chenarides, Arizona State University  Co-authors: Zachariah Rutledge, Timothy J. Richards, and John Pender  **Research Panel 4 – *The Changing Role of Dollar Stores***  *Dollar stores & supermarkets in small towns: changes in the retail landscape* Presenter: Jerry Shannon, University of Georgia Co-author: Jennifer Mapes  *What Happens When Dollar Stores Accept EBT?* Presenter: Anne Byrne, Economic Research Service Co-authors: Xiao Dong, Jessie Handbury, Erik James, and Katherine Meckel  *The Healthfulness of Foods Purchased at Dollar Stores, 2008 - 2020* Presenter: Wenhui Feng, Tufts University Co-authors: Elina Page, Bangyao Sun, and Sean Cash  **Research Panel 5 - *Community Perspectives***  *Characterizing the Dollar Store Landscape in Atlanta* Presenter: Samantha Sundermeir, Johns Hopkins Bloomberg School of Public Health Co-authors: Emma C. Lewis, Lisa Poirier, Megan R. Winkler, and Joel Gittelsohn  *Stretching your dollar: experiences among dollar store shoppers in Fresno and Boston during the first year of the COVID-19 pandemic* Presenter: Alexandria Schmall, Tufts University Friedman School of Nutrition Science and Policy Co-authors: Sara C. Folta, Wenhui Feng, and Sean B. Cash  *Dollar Stores and the Food Environment: Perspectives from the Community* Presenter: Sara John, Center for Science in the Public Interest Co-authors: Matt George, Jessa Scott-Johnson, Claudia Malloy, and Ashley Hickson |
| --- |

**Posters:**

*Dollar Store Market Expansion*
Lauren Chenarides, Arizona State University and Alexandra E. Hill, Colorado State University

*Virtual Reality and Date Label Education in Dollar General Stores*
Priscilla Connors and Audon Archibald, University of North Texas

*Integrating Sociological, Psychological, and Health Factors into a System Dynamics Model of Food Insecurity in Suburban Regions: The Role of Community Organizations, Schools, and Dollar Stores*
Meveryn Chua, William and Mary School of Education

*Expansion of Dollar Stores and its Effects on Food Retailers' Entry and Exit and Food Prices*
Metin Çakır, University of Minnesota, Lauren Chenarides, Arizona State University, Qingxiao Li, University of Minnesota, and Timothy J. Richards, Arizona State University

*Dollar Tree and Food Access*
Fangruo (Ingrid) Zhou and Yongyi Pan, Tufts University Friedman School of Nutrition Science and Policy

*Pricing Strategies and Retail Competition at Dollar Stores*
Camilla Schneier, Fern Ramoutar ,and Yixin Sun, The University of Chicago Booth School of Business
